# Intensive Circulation of Japanese Encephalitis Virus in Peri-urban Sentinel Pigs near Phnom Penh, Cambodia

**DOI:** 10.1371/journal.pntd.0005149

**Published:** 2016-12-07

**Authors:** Julien Cappelle, Veasna Duong, Long Pring, Lida Kong, Maud Yakovleff, Didot Budi Prasetyo, Borin Peng, Rithy Choeung, Raphaël Duboz, Sivuth Ong, San Sorn, Philippe Dussart, Arnaud Tarantola, Philippe Buchy, Véronique Chevalier

**Affiliations:** 1 CIRAD-ES, UPR AGIRs, Montpellier, France; 2 Institut Pasteur du Cambodge, Epidemiology and Public Health Unit, Phnom Penh, Cambodia; 3 Institut Pasteur du Cambodge, Virology Unit, Phnom Penh, Cambodia; 4 Royal University of Agriculture, Phnom Penh, Cambodia; 5 United States Naval Medical Research Unit 2, Phnom Penh, Cambodia; 6 National Veterinary Research Institute, Phnom Penh, Cambodia; 7 GlaxoSmithKline Vaccines R&D, Singapore, Singapore; Centers for Disease Control and Prevention, Puerto Rico, UNITED STATES

## Abstract

Despite the increased use of vaccination in several Asian countries, Japanese Encephalitis (JE) remains the most important cause of viral encephalitis in Asia in humans with an estimated 68,000 cases annually. Considered a rural disease occurring mainly in paddy-field dominated landscapes where pigs are amplifying hosts, JE may nevertheless circulate in a wider range of environment given the diversity of its potential hosts and vectors. The main objective of this study was to assess the intensity of JE transmission to pigs in a peri-urban environment in the outskirt of Phnom Penh, Cambodia. We estimated the force of JE infection in two cohorts of 15 sentinel pigs by fitting a generalised linear model on seroprevalence monitoring data observed during two four-month periods in 2014. Our results provide evidence for intensive circulation of JE virus in a periurban area near Phnom Penh, the capital and most populated city of Cambodia. Understanding JE virus transmission in different environments is important for planning JE virus control in the long term and is also an interesting model to study the complexity of vector-borne diseases. Collecting quantitative data such as the force of infection will help calibrate epidemiological model that can be used to better understand complex vector-borne disease epidemiological cycles.

## Author Summary

Japanese Encephalitis Virus (JEV) is the most important cause of viral encephalitis in Asia in humans with an estimated 68,000 cases annually. The disease is considered a mainly rural one because it occurs mainly in rural areas dominated by paddy fields where the main mosquito species vector of JEV breed. However, other mosquito species, breeding in urban areas, and a large range of animal hosts can play a role in the transmission of JEV, and JEV could therefore be transmitted in peri-urban and urban areas. Our results show an intensive circulation of JEV in sentinel pigs in a peri-urban area of Phnom Penh Cambodia at two different periods of the year. It shows the potential for JEV to circulate in a large range of landscapes and suggest that JEV control should not be limited to rural areas and that JEV may have the potential to emerge and be and be maintained in new areas.

## Introduction

Despite the increased use of vaccination in several Asian countries, Japanese Encephalitis (JE) remains the most important cause of viral encephalitis in Asia in humans [1–3]. A recent review based on updated incidence data estimated that 68,000 JE cases occurred annually in the 24 JE-endemic countries, for an estimated incidence of 1.8 case per 100°000 people overall [1]. Half of these cases occur in China where expanding vaccination programs should dramatically decrease the incidence of JE in the future. One-fifth occur in areas with no or minimal JE vaccination programme such as Cambodia [1].

Cambodia is a JE high-incidence country with a nascent vaccination programme that should develop into a national program in the coming years [4]. A sentinel surveillance study on Japanese encephalitis in six Cambodian hospitals estimated the clinically-declared JE incidence in 2007 in the country at 11.1 cases per 100 000 children under 15 years of age [4].

The epidemiological cycle of JE is complex with different potential host and vector species. JE is considered a predominantly rural zoonosis with a wild cycle involving aquatic birds and *Culex* mosquitoes and a domestic cycle where pigs are amplifier hosts [5,6]. This classical description of JE in which wild ardeids are considered the main reservoir of JE dates back to the 1950s and the first extensive studies of JE epidemiology in Japan [7]. The proximity to irrigated land and in particular paddy fields where JE vectors can breed and the presence of pigs, typical features of most rural areas in Cambodia and other East and South-East Asian countries, have been identified as JE risk factors [8–11].

Several *Culex* species have been identified as potential JE vectors [5]. The main vectors such as *Culex tritaeniorhynchus* breed mostly in rural settings, however, other species like *Culex quinquefasciatus*, an anthropophilic species, could play an a role in JE transmission in periurban or urban areas [12,13]. Beyond the aquatic wild birds traditionally suspected to be the main reservoir [5,14], several host species are also thought to be able to play a role in the transmission of the virus such as poultry or non-aquatic wild birds such as passerine birds that experimentally show sufficient viremia to allow virus transmission [15–17]. This means that JE could be transmitted and even maintained in a wide range of environments beyond the typical rural, paddy-fields dominated landscape.

JE epidemiology should be rethought depending on the different environments and hosts [17]. With JE expanding [18,19], it is important to understand the range of eco-epidemiological systems in which it could be maintained and transmitted to humans, especially in peri-urban or even urban areas where a growing part of the world population is living. This peri-urban and urban circulation has been observed in Southeast Asia where peri-urban human JE cases have been observed in Bangkok, Thailand and Can Tho, Vietnam [3,20]. Similarly, JE may still be actively transmitted in the peripheral part of the Singapore island despite abolishment of pig farming [21].

The main objective of this study was to assess the intensity of JEV transmission to pigs in a periurban environment in the outskirt of Phnom Penh, Cambodia. Specifically, we estimated the force of JEV infection in two cohorts of sentinel pigs by detecting anti-JEV IgG antibodies from the end of the hot dry season to the beginning of the rainy season (from April to July) and subsequently from the peak of the rainy season to the beginning of the cool dry season (from September to January). Concomitantly, we captured mosquitoes and tested them for JEV by qRT-PCR during the pig sampling periods to infer JEV potential vector species in this area.

## Results

### Sentinel pigs

**Fig 1:**
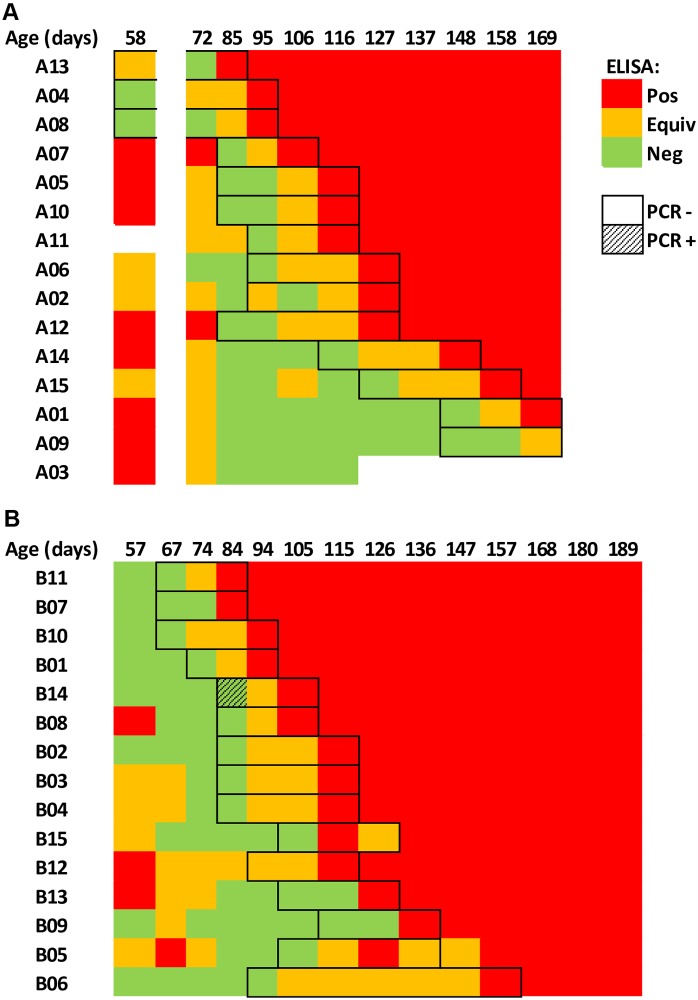
Summary of the ELISA and RT-PCR results of pig sera tested for Japanese encephalitis antibodies and virus RNA.

**Fig 2:**
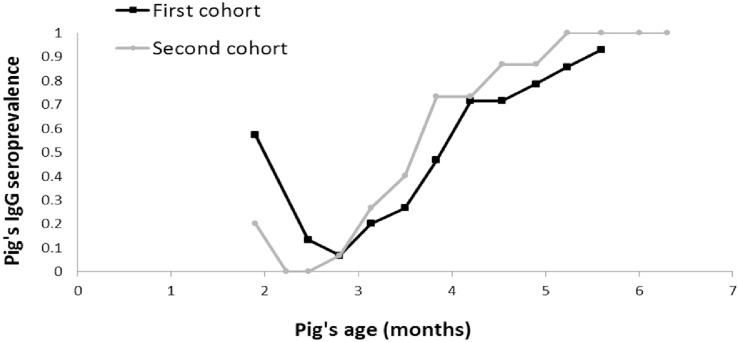
Evolution of the pigs’ IgG seroprevalence with age.

Of 29 pigs that remained in the study (Pig A03 died of unknown cause during the study, its last blood sample tested negative for JE antibodies), 28 seroconverted during the study period (Fig 1). Test results of the last collected serum were equivocal in the 29^th^ pig (A09) (Fig 1). Some pigs still had maternal antibodies at the age of two months, but all of them had become seronegative by the age of three months before rapidly seroconverting again between the age of three months and six months during both study periods (Fig 2). All seroconverted pigs were confirmed positive to JE by SNT. No clinical signs were recorded in any of the pigs during the study (except for pig A03).

qRT-PCR-screened blood samples taken before seroconversion (n = 106) were negative in all but one pig (pig B14 on 29/09/2014 (fourth blood sample) (Fig 1). The virus isolation attempt was not successful after three passages. The sample was confirmed positive by amplicon sequencing of NS3 gene.

### Estimation of the force of JEV infection in the sentinel pigs

**Fig 3:**
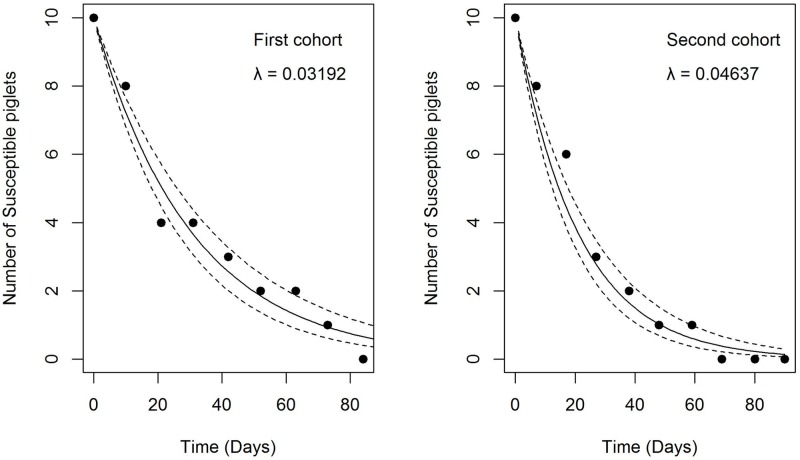
Estimated FOI (λ) for period 1 (n = 10 pigs) and period 2 (n = 10 pigs). The number of susceptible pigs in our sentinel cohorts is shown as points, the fitted model is shown as a solid line with standard deviation as dashed lines.

For the first cohort, we set May 6, 2014 (date of the third blood sample) as the starting date with ten susceptible pigs (Pig A03 was removed from the study as it died before seroconverting). The model estimated a FOI of 0.03192/day (sd = 0.005622/days), meaning that during that period, a susceptible pig had a 3.19% probability of acquiring JEV infection each day. For the second cohort, we set September 12, 2014 (date of the second blood sample) as the starting date with ten susceptible pigs. The model estimated a FOI of 0.04637/days (sd = 0.007973/day), meaning that a susceptible pig had a daily probability of 4.64% of acquiring JEV infection during that period. Fig 3 shows the fitted model over our data.

### Mosquitoes

**Fig 4:**
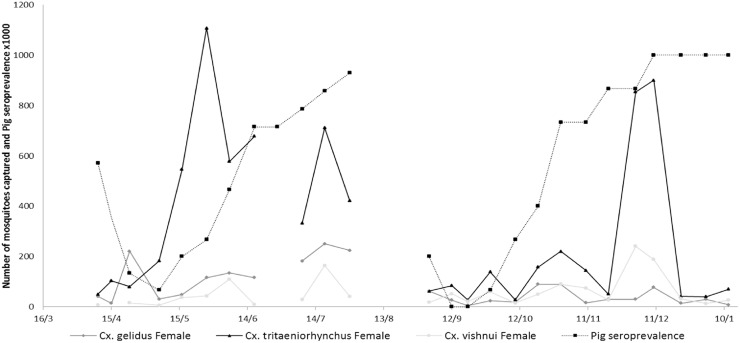
Evolution of the number of trapped mosquitoes and pig seroconversion dynamic during the two survey periods.

A total of 11,078 mosquitoes were captured, 6,692 during the 11 capture sessions between April and July and 4,386 during the 14 capture sessions between September and January. Table 1 shows a summary of the mosquito species captured during the study, detailed results are available in S1 Table. *Culex tritaeniorhynchus* was the most abundant species with around 2/3 of the mosquitoes captured during both study periods, followed by *Culex gelidus* in April-July and *Culex vishnui* in September-January (Table 1). Around 1% of the mosquitoes captured were *Culex quinquefasciatus*. The number of mosquitoes captured varied greatly during the study with apparent peak of mosquito’s abundance in May, July and December (Fig 4). A total of 1,171 pools were screened for JEV using qRT-PCR. Only 1 pool of *Culex tritaeniorhynchus*, captured on 12/09/2014 was found positive, i.e. a minimum infection rate (MIR) of 11.9/ 1,000 for *Culex tritaeniorhynchus* females for this night of capture, a MIR of 0.13/ 1,000 for *Culex tritaeniorhynchus* females over the whole study and MIR of 0.091/ 1,000 for females from all species over the whole study.

**Table 1:** Summary of the number of mosquitoes captured per species.

Species	Number captured			Number of pools
	Apr-Jul	Sep-Jan	Total	
*Cx*. *tritaeniorhynchus* Female	4791 (71.6%)	2819 (64.3%)	7610 (68.7%)	766
*Cx*. *gelidus* Female	1376 (20.6%)	521 (11.9%)	1897 (17.1%)	199
*Cx*. *vishnui* Female	462 (6.9%)	908 (20.7%)	1370 (12.4%)	144
*Cx*. *quinquefasciatus* Female	16 (0.2%)	87 (2.0%)	103 (0.9%)	21
Other	47 (0.7%)	51 (1.2%)	98 (0.9%)	41
Total	6692	4386	11078	1171

## Discussion

Our results provide evidence for intensive circulation of JEV in a periurban area near Phnom Penh, the capital and most populated city of Cambodia. Among 29 pigs, 28 (96.6%) had seroconverted before the age of six months, and the last serological result of the 29^th^ pig was equivocal, suggesting that it was seroconverting as most other pigs had a serum tested equivocal prior to the seroconversion. This is in line with results observed in rural Cambodia where 95.2% of the pigs older than 6 months were tested seropositive for JEV by IgG ELISA and hemagglutination inhibition tests [22]. This suggests that JEV circulation in periurban areas near Phnom Penh may be as intensive as in rural Cambodia. This intensive JEV circulation in pigs was also observed in a urban environment in the city of Can Tho in Vietnam [20], pointing out the importance of taking into account the risk of JEV transmission in urban and periurban areas, and not in the typical rural environment only. Populations living in such periurban areas are growing and they should be enrolled in national JEV control programmes.

Our protocol allowed us to estimate the FOI of JEV in the sentinel pig population. Results from the two cohorts were close and may suggest a lack of seasonality in transmission but our data are not sufficient to support this point. Indeed, it was not possible to use a simple modified Welch t-test to compare the two FOI because they were estimated using a maximum likelihood approach and the distribution of such estimators can only be approximated to normality when the information about the studied population is almost exhaustive, i.e. for large sample sizes [23]. Furthermore, the relatively large standard deviations associated with the FOI (coefficients of variation of 17.6% and 17.2%) are likely due to our limited sample size and would prevent any interpretation of a non-significant difference between the FOI estimated for the two cohorts. This is, to our knowledge, the first estimation in East and Southeast Asia of the force of JEV infection in pigs. It was estimated in Bangladesh at 20% per year [24], which is considerably lower than the FOI estimated in our periurban study area (3–5% per day). This difference may translate a different combination of hosts, vectors and agricultural practices in the two areas, pointing out the importance of taking into account these parameters when planning control programs [17]. In the absence of wild waterbirds in periurban and urban areas, domestic and peridomestic species such as passerine birds may play a role in the transmission and the maintenance of the virus as suggested by the JEV viremia experimentally observed in poultry [15] and in several native North American passerine species [16].

In terms of vectors, *Culex tritaeniorhynchus*—a species mostly considered rural because it breeds in fresh water such as flooded paddy fields—was the most abundant mosquito species captured, while *Culex quinquefasciatus*, a “domestic” species, accounted for around 1% only of the total number of mosquitoes captured. As our study was set in a periurban area we could have expected a more balanced proportion of rural and domestic vectors. The location of our traps, close to the pigs, may have influenced our results and led to capture more *Culex tritaeniorhynchus*, a predominantly zoophilic species, than *Culex quinquefasciatus*, a more anthropopihlic species. These results suggest that *Culex tritaeniorhynchus* can be abundant at least in small parts of periurban areas and play a major role in the intensive circulation of JEV, as observed in the urban area of Can Tho city in Vietnam [20]. Intensive circulation of JEV in other urban and periurban areas may then be dependent on the presence of a vector as competent as *Culex tritaeniorhynchus*. Countries beyond JEV geographical distribution, where *Culex tritaeniorhynchus* is present, should then implement JEV surveillance in both rural and peri-urban areas [25–27].

Despite an intensive circulation of JEV detected in pigs, only one pool of *Culex tritaeniorhynchus* tested positive for JEV by qRT-PCR. This low detection rate of JEV in mosquitoes may be related to an actual low infection rate in mosquitoes, as observed for other vector-borne diseases including other flavivirus closely related to JEV such as West Nile virus [28,29], and/or to the dilution effect resulting from pooling the mosquitoes before testing with a molecular method that has its own limit of detection. The MIR of 0.091/ 1,000 for females from all species over our whole study is low compared to MIR in the range of 1–1.2/ 1,000 for a JEV study in Can Tho city [20] and two West Nile studies in Florida and Puerto Rico [28,29] but is similar to previous studies on JEV in rural areas of Can Tho province (MIR of 0.05 / 1,000) and in suburban Bangkok (MIR of 0.046 / 1,000) [30,31]. An actual low infection rate of JEV in mosquitoes despite an intensive circulation in pigs could be consistent with the existence of a direct transmission of JEV between pigs as suggested by the results of a recent experimental study showing that oro-nasal virus excretion could last -5-6 days in pigs [32]. The FOI we are estimating in this study would then result from a combination of a vector-borne and a “within-pen” direct transmission. Based on the same set of data, we are currently developing dynamic models to quantify the relative importance of the different transmission routes.

Control of JEV in humans has successfully been implemented in several Asian countries over the past decades by introducing vaccination [33]. Mass vaccination campaigns have dramatically decreased the number of clinical acute encephalitis in countries like Japan and South Korea after their introduction [33,34]. Since humans are dead-end hosts due to very low viremia, their vaccination does not disrupt the transmission of JEV, and given the complexity of JEV epidemiological cycle, eradicating the disease does not seem realistic. However, other control measures can be combined with human vaccination (or when vaccination is not available) to protect humans, which may be especially important if vaccination become less efficient in the future against emergent genotypes [35]. Vaccination can also protect pigs from abortions or orchitis. In Cambodia and in endemic areas with an intensive JEV circulation in general, JEV has little to no impact on pig production since most pigs get infected prior to reaching sexual maturity, as observed in our study or in South Vietnam [20]. But in epidemic areas such as North Vietnam or China, JE is an animal disease and control measures such as vaccination of reproductive pigs can be used. Estimating key parameters such as the force of infection to calibrate models of JEV transmission may then be used to test different measures (i.e. pig vaccination, banning pig farming near populated areas, rice flooding management) and optimize JE control according to the local situation in humans and animals. The impact of pig vaccination was for example predicted as an interesting JE control tool in Bangladesh for both animals and humans [24].

Beyond estimating key transmission parameters, the surveillance of JEV with sentinel pigs could also be used to detect JEV emergence. Historically, JEV spread geographically in Asia from the Indonesia-Malaysia region [18,36] with a recent emergence in Australia [37]. It may potentially emerge in a diversity of ecosystems including Africa or Europe. As a matter of fact, JEV-RNA like sequences were detected in Italy and *Culex tritaeniorhynchus* established in Greek paddy fields [26,38]. Confirming JEV infection in humans is challenging: direct detection methods such as viral isolation or qRT-PCR have low sensitivity because of transient, early viraemia and diagnosis of JEV infection by IgM detection might be misled by antigenic cross-reaction and by actual secretion of anti-JEV IgM during another neurological infection in patients previously immunised against JEV [39]. Detecting JEV emergence may then be easier in pigs. A first step for detecting JEV emergence could be a routine serological surveillance of pig populations at slaughterhouses in risky areas, followed by the implementation of sentinel pig surveillance in case of positive results. This would help to confirm the emergence, to characterise the virus in pigs and vectors and to quantify the transmission in the emergence area.

More generally, with several species of mosquitoes—mostly from the *Culex* genus,—known as JEV vectors [5] and a large diversity of potential hosts, understanding JEV transmission in different environments is important for planning JEV control in the long term and is also an interesting model to study the complexity of vector-borne diseases. Measuring quantitative data such as the force of infection will help calibrate epidemiological model that can be used to better understand complex vector-borne disease epidemiological cycles and test different strategies of control.

## Methods

### Ethics statement

During this study, we followed the World Animal Health Organisation (OIE) guiding principles on animal welfare included in the OIE terrestrial Code, Chapter 7.8 “Use of Animals in research and education” [40]. In particular, intervals between sampling sessions were 10 days to limit the stress resulting from handling and sampling. At the beginning of the study the pigs were not sampled for two weeks in order to let them acclimate to their new environment. They were separated in three groups of five individuals in separated pens.

### Study site

**Fig 5:**
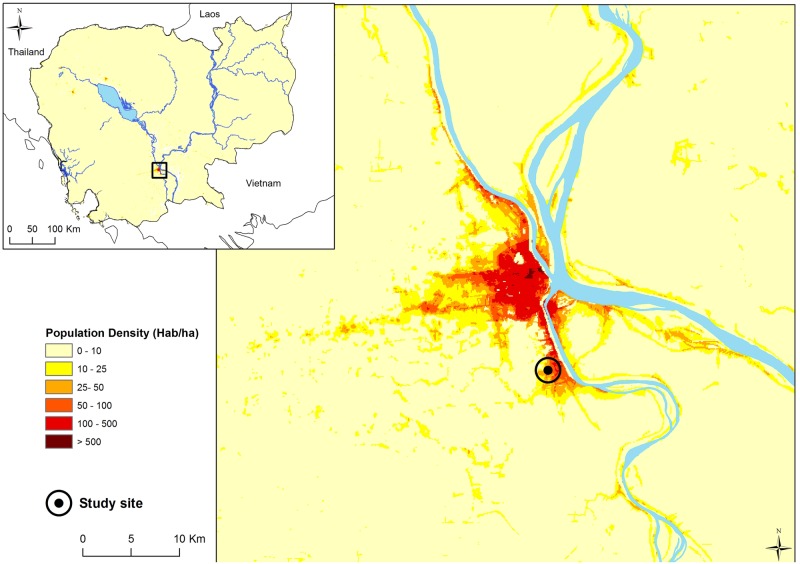
Location of the study site in a periurban area of Phnom Penh, Cambodia. Population density data are extracted from the WorldPop project [41].

The study was set in the city of Ta Khmau, in a periurban area located 10 km from the center of Phnom Penh (11.4739°N, 104.9376°E), at the interface between a densely populated urban area and a rural landscape dominated by cultivated areas (Fig 5).

### Sentinel pigs and blood sampling

Two cohorts of 15 pigs were successively monitored from April to July 2014 and from September 2014 to January 2015. Pigs were bought at the age of six weeks and kept in a backyard where no domestic animals were usually raised. The pigs were individually identified with ear tags. Blood samples were collected every ten days on every pig from the age of 2 months, when maternal immunity is waning, to the age of six months when pigs are usually sent to the slaughterhouse.

Pigs of the first cohort, tagged A01 to A15 were born on February 10, 2014, and sampled 11 times from April 9 to July 29, 2014. Pigs of the second cohort, tagged B01 to B15 were born on July 7, 2014, and sampled 14 times from September the 2^nd^ to January 12, 2015.

### Sentinel pigs serum analysis

Sera were tested for JEV IgG using an ELISA test adapted from Dong Kun Yang et al [42]. The value of the background absorbance was subtracted from the signal value of all the test reading. The cut off value was calculated **based on the mean of the three negative controls (NC): positive if the sample optical density (OD) > 4x mean NC, negative if the sample OD < 3 mean NC, and equivocal if the sample OD is within 3x mean NC and 4x mean NC**. At the end of the study, the last serum sample of each pig underwent serum neutralization testing (SNT) for JEV [43]. BHK-21 cells (ATCC, CCL-10) were initially inoculated at 1 × 10^6^ cells/well in six-well tissue culture plates and propagated for 24 hours at 37°C in a CO_2_ incubator. Serum samples were inactivated for 30 minutes in a 56°C water-bath and serially diluted ten-fold from 1:10 to 1:1000 in Dulbecco's Modified Eagle Medium (DMEM) containing 10% fetal bovine serum (FBS). A 100-μL aliquot of JEV (JEV SA 14-14-2) with 60 plaque-forming units (pfu) was mixed with equal volumes of diluted serum samples and incubated for 1 hour at 37°C. Each virus/serum mixture (total volume 200 μL) was inoculated onto the BHK-21 cell monolayer after draining the culture medium and was allowed to settle for 1 hour at 37°C in a CO_2_ incubator. The mixture was removed from the cell monolayer and each well washed once with phosphate-buffered saline (PBS). Then 4 mL of pre-warmed overlay medium consisting of 3% Carboxymethyl cellulose (Sigma, Cat. C4888) and 0.9% NaCl (Sigma, Cat. S6191) and 3% FBS in DMEM were poured onto each well. The plates were placed in a CO_2_ incubator and the overlay medium was removed five days after inoculation. Each well was carefully washed two time with PBS and was stained with 0.1% Naphthol Blue Black (Sigma, Cat. N3393), 25% Isopropanol (Sigma, Cat. I9516) and 10% Acetic acide (Sigma, Cat. 320099) for 30 minutes. Plate wells were slowly washed and dried and the plaques were counted. The neutralizing antibody titer (PRNT_50_) was defined as the reciprocal of the last serum dilution that showed 50% or more plaque reduction compared with the plaque counts in the virus-only control well. PRNT_50_ titres ≥1:20 were considered positive.

Because IgG antibodies can start to be detected up to several weeks after the infection in pigs [44], serum samples collected within two weeks prior to the presumed seroconversion date of each pig were tested by quantitative reverse transcriptase polymerase chain reaction (qRT-PCR) to detect JEV RNA [45]. The limit of detection of the assay was 10 copy of RNA/reaction. Conventional PCR using primers targeting NS3 region (S2 Table) was used on positive samples by qRT-PCR and the PCR products were sequenced for confirmation [46].

### Mosquitoes

One home-made CDC light-trap was placed in the pig open-building during the night preceding each blood sampling of the two cohorts [47]. Mosquitoes captured were identified using a Southeast Asia identification key [48,49]. Mosquitoes were counted and pooled by groups of ten individuals of the same species and the same night of capture and subsequently screened for JEV by qRT-PCR.

### Estimation of the force of infection of JEV in the sentinel pigs

The monitoring of the serological status of the sentinel pigs enabled us to estimate the Force Of Infection (FOI) of JEV for each cohort. FOI is the instantaneous probability of a susceptible individual to become infected over a short period of time. As proposed by Heisey et al [50], FOI can be expressed as a function of the number of susceptible individuals over time:

dS(t)/dt=−λS(t) (1)

With S(t) the number of susceptible individuals at time t, and λ the force of infection.

(Eq 1) has for solution:

$$\text{S}\left(\text{t}\right){\text{ =S}}_{\text{0}}*\text{ exp (}-\lambda \text{t)}$$ (2)

With S_0_ the number of susceptible individuals at t = 0. (Eq 2) can be linearized as:

$$\text{ln }\left(\text{S}\left(\text{t}\right)\right)\text{ = ln }\left({\text{S}}_{\text{0}}\right)-\lambda \text{t}$$ (3)

In (Eq 3), FOI can be estimated with a generalized linear model if information is available on the evolution over time of the number of susceptible individuals from a starting date (t = 0). We estimated the FOI by fitting a generalized linear model to our data depicting the transition of susceptible pigs (tested negative with our ELISA test) into non-susceptible pigs (tested positive with our ELISA test). For each cohort we used as the starting date (t = 0) the first date with the highest number of susceptible pigs in order to increase precision and a time step (dt) of 1 day. Using this method, we assumed that the FOI was constant over each study period. We used the glm() function in the R software [51]. For our estimation of the FOI, we considered the pigs tested equivocal with the ELISA test as non-susceptible.

### Statement

"The views expressed in this article are those of the author and do not necessarily reflect the official policy or position of the Department of Navy, Department of Defense, nor the U.S. Government."

## Supporting Information

S1 Table Detailed results of the mosquito trapping and testing. (PDF)

S2 Table Oligonucleotide primers for qRT-PCR amplification of JEV complete genome. (PDF)
