# Supplementary material for: Intensive Circulation of Japanese Encephalitis Virus in Peri-urban Sentinel Pigs near Phnom Penh, Cambodia
Source: PLoS Negl Trop Dis. 2016 Dec 7;10(12):e0005149. doi: 10.1371/journal.pntd.0005149 (PMC5142769; doi:10.1371/journal.pntd.0005149)
Supplement: S1 Table — (PDF) [file pntd.0005149.s001.pdf]

S1 Table. Detailed results of the mosquito trapping and testing

Date	*Species*	*Sex*	*Number*	*N Pool*	*JEV PCR result*
09/04/2014	Cx.gelidus	F	41	5	Negative
09/04/2014	Cx.vishnui group	F	8	1	Negative
09/04/2014	Cx.tritaeniorhynchus	F	50	5	Negative
15/04/2014	Cx.gelidus	F	14	2	Negative
15/04/2014	Cx.tritaeniorhynchus	F	104	11	Negative
15/04/2014	Other spp	F	1	1	Negative
23/04/2014	Cx.gelidus	F	220	22	Negative
23/04/2014	Cx.vishnui group	F	16	2	Negative
23/04/2014	Cx.tritaeniorhynchus	F	80	8	Negative
23/04/2014	Other spp	F	4	1	Negative
06/05/2014	Cx.gelidus	F	31	4	Negative
06/05/2014	Cx.vishnui group	F	6	1	Negative
06/05/2014	Cx.tritaeniorhynchus	F	183	19	Negative
06/05/2014	Other spp	F	2	1	Negative
20/05/2014	Cx.gelidus	F	48	5	Negative
20/05/2014	Cx.vishnui group	F	37	4	Negative
20/05/2014	Cx.tritaeniorhynchus	F	546	55	Negative
20/05/2014	Cx.quinquefasciatus	F	1	1	Negative
20/05/2014	Mansonia spp	F	1	1	Negative
20/05/2014	Other spp	F	1	1	Negative
27/05/2014	Cx.gelidus	F	116	12	Negative
27/05/2014	Cx.gelidus	M	2	1	Negative
27/05/2014	Cx.vishnui group	F	43	5	Negative

27/05/2014	Cx.tritaeniorhynchus	F	1107	111	Negative
27/05/2014	Other spp	M	3	1	Negative
06/06/2014	Cx.gelidus	F	134	14	Negative
06/06/2014	Cx.vishnui group	F	110	11	Negative
06/06/2014	Cx.tritaeniorhynchus	F	578	58	Negative
06/06/2014	Mansonia spp	F	2	1	Negative
06/06/2014	Other spp	F	1	1	Negative
12/06/2014	Cx.gelidus	F	116	12	Negative
12/06/2014	Cx.vishnui group	F	9	1	Negative
12/06/2014	Cx.tritaeniorhynchus	F	678	68	Negative
12/06/2014	Cx.quinquefasciatus	F	3	1	Negative
12/06/2014	Mansonia spp	F	1	1	Negative
12/06/2014	Mansonia spp	M	1	1	Negative
12/06/2014	Other spp	F	3	1	Negative
12/06/2014	Other spp	M	2	1	Negative
27/06/2014	Cx.gelidus	F	1	1	Negative
27/06/2014	Cx.vishnui group	F	3	1	Negative
27/06/2014	Cx.tritaeniorhynchus	F	3	1	Negative
08/07/2014	Cx.gelidus	F	182	18	Negative
08/07/2014	Cx.vishnui group	F	28	3	Negative
08/07/2014	Cx.tritaeniorhynchus	F	332	33	Negative
08/07/2014	Cx.quinquefasciatus	F	2	1	Negative
18/07/2014	Cx.gelidus	F	250	25	Negative
18/07/2014	Cx.gelidus	M	1	1	Negative
18/07/2014	Cx.vishnui group	F	164	17	Negative

18/07/2014	Cx.tritaeniorhynchus	F	711	71	Negative
18/07/2014	Cx.quinquefasciatus	F	3	1	Negative
29/07/2014	Cx.gelidus	F	224	23	Negative
29/07/2014	Cx.gelidus	M	4	1	Negative
29/07/2014	Cx.vishnui group	F	41	4	Negative
29/07/2014	Cx.tritaeniorhynchus	F	422	42	Negative
29/07/2014	Cx.quinquefasciatus	F	7	1	Negative
29/07/2014	Cx.quinquefasciatus	M	13	2	Negative
29/07/2014	Cx. fuscocephala	F	1	1	Negative
29/07/2014	Mansonia spp	F	1	1	Negative
29/07/2014	Other spp	F	1	1	Negative
29/07/2014	Anopheles spp	F	2	1	Negative
02/09/2014	Cx.gelidus	F	62	6	Negative
02/09/2014	Cx.tritaeniorhynchus	F	63	6	Negative
02/09/2014	Cx.tritaeniorhynchus	M	4	1	Negative
02/09/2014	Cx.vishnui group	F	18	2	Negative
02/09/2014	Cx.vishnui group	M	6	1	Negative
02/09/2014	Cx.quinquefasciatus	F	2	1	Negative
12/09/2014	Cx.gelidus	F	26	3	Negative
**12/09/2014**	**Cx.tritaeniorhynchus**	**F**	**84**	**9**	**1 pool Positive**
12/09/2014	Cx.vishnui group	F	52	5	Negative
12/09/2014	Cx.quinquefasciatus	F	1	1	Negative
19/09/2014	Cx.gelidus	F	5	1	Negative
19/09/2014	Cx.tritaeniorhynchus	F	27	3	Negative
19/09/2014	Cx.tritaeniorhynchus	M	1	1	Negative
19/09/2014	Cx.vishnui group	F	21	2	Negative

19/09/2014	Cx.quinquefasciatus	F	2	1	Negative
19/09/2014	Cx.quinquefasciatus	M	2	1	Negative
19/09/2014	Mansonia spp	F	1	1	Negative
19/09/2014	Anopheles spp	F	1	1	Negative
29/09/2014	Cx.gelidus	F	24	3	Negative
29/09/2014	Cx.gelidus	M	1	1	Negative
29/09/2014	Cx.tritaeniorhynchus	F	139	14	Negative
29/09/2014	Cx.vishnui group	F	60	6	Negative
29/09/2014	Cx.quinquefasciatus	F	5	1	Negative
29/09/2014	Cx.quinquefasciatus	M	9	1	Negative
29/09/2014	Mansonia spp	F	1	1	Negative
29/09/2014	Other spp	F	1	1	Negative
29/09/2014	Other spp	M	1	1	Negative
29/09/2014	Anopheles spp	F	1	1	Negative
10/10/2014	Cx.gelidus	F	19	3	Negative
10/10/2014	Cx.tritaeniorhynchus	F	28	3	Negative
10/10/2014	Cx.vishnui group	F	15	2	Negative
10/10/2014	Cx.quinquefasciatus	F	10	1	Negative
10/10/2014	Cx.quinquefasciatus	M	2	1	Negative
20/10/2014	Cx.gelidus	F	90	9	Negative
20/10/2014	Cx.tritaeniorhynchus	F	158	16	Negative
20/10/2014	Cx.vishnui group	F	50	5	Negative
20/10/2014	Cx.quinquefasciatus	F	4	1	Negative
20/10/2014	Cx.quinquefasciatus	M	2	1	Negative
20/10/2014	Mansonia spp	F	2	1	Negative
20/10/2014	Other spp	F	1	1	Negative

30/10/2014	Cx.gelidus	F	90	9	Negative
30/10/2014	Cx.tritaeniorhynchus	F	220	22	Negative
30/10/2014	Cx.vishnui group	F	90	9	Negative
30/10/2014	Cx.quinquefasciatus	F	19	2	Negative
30/10/2014	Anopheles spp	F	10	1	Negative
30/10/2014	Mansonia spp	F	3	1	Negative
30/10/2014	Other spp	F	1	1	Negative
10/11/2014	Cx.gelidus	F	16	2	Negative
10/11/2014	Cx.vishnui group	F	75	8	Negative
10/11/2014	Cx.tritaeniorhynchus	F	145	15	Negative
10/11/2014	Cx.quinquefasciatus	F	5	1	Negative
10/11/2014	Cx. fuscocephala	F	1	1	Negative
20/11/2014	Cx.gelidus	F	30	3	Negative
20/11/2014	Cx.tritaeniorhynchus	F	51	5	Negative
20/11/2014	Cx.vishnui group	F	27	3	Negative
20/11/2014	Cx.quinquefasciatus	F	2	1	Negative
02/12/2014	Cx.gelidus	F	30	3	Negative
02/12/2014	Cx.vishnui group	F	241	24	Negative
02/12/2014	Cx.tritaeniorhynchus	F	853	86	Negative
02/12/2014	Cx.quinquefasciatus	F	11	1	Negative
10/12/20014	Cx.gelidus	F	77	8	Negative
10/12/20014	Cx.vishnui group	F	188	19	Negative
10/12/20014	Cx.quinquefasciatus	F	4	1	Negative
10/12/20014	Cx.tritaeniorhynchus	F	900	90	Negative
22/12/2014	Cx.gelidus	F	14	2	Negative

22/12/2014	Cx.tritaeniorhynchus	F	41	4	Negative
22/12/2014	Cx.vishnui group	F	32	4	Negative
22/12/2014	Cx.quinquefasciatus	F	2	1	Negative
02/01/2015	Cx.gelidus	F	30	3	Negative
02/01/2015	Cx.tritaeniorhynchus	F	40	4	Negative
02/01/2015	Cx.vishnui group	F	13	2	Negative
02/01/2015	Cx.quinquefasciatus	F	4	1	Negative
12/01/2015	Cx.gelidus	F	8	1	Negative
12/01/2015	Cx.tritaeniorhynchus	F	70	7	Negative
12/01/2015	Cx.vishnui group	F	26	3	Negative
12/01/2015	Cx.quinquefasciatus	F	16	2	Negative
