# Supplementary material for: Intensive Circulation of Japanese Encephalitis Virus in Peri-urban Sentinel Pigs near Phnom Penh, Cambodia
Source: PLoS Negl Trop Dis. 2016 Dec 7;10(12):e0005149. doi: 10.1371/journal.pntd.0005149 (PMC5142769; doi:10.1371/journal.pntd.0005149)
Supplement: S2 Table — (PDF) [file pntd.0005149.s002.pdf]

S2 Table: Oligonucleotide primers for qRT-PCR amplification of JEV complete genome

Primer name	Oligonucleotide sequence (5-3)	Orientation
JEN(1-20)*	TGTGTCAACTTCTTGGCTTA	Sense
JEN(520-539)	GCTTGCAATGTCCGTGTTGT	Antisense
JEM(429-448)	ATCATGTGGCTCGCAAGCTT	Sense
JEM(1,029-1,048)	TCCTTCTAGCACCAAGTACA	Antisense
JEE(960-979)	GTCGCTCCGGCTTACAGTTT	Sense
JEE(2,482-2,501)	GATGTCAATGGCACAGCCGT	Antisense
JENS(2,478-2,498)	GACACTGGATGTGCCATTGAC	Sense
JENS(3,512-3,533)	AGCATCAACCTGTGATCTGACG	Antisense
JENS(3,481-3,500)	TCAGACCTGTTAGGCATGAT	Sense
JENS(4,421-4,440)	CTAGCCTCCGGCTGCTTCCT	Antisense
JENS(4,381-4,400)	GGGCTGCCGATATCAGCTGG	Sense
JENS(5,351-5,370)	TTCCCTGATGCTCCCTCTGC	Antisense
JENS(5,313-5,322)	TTGAGAGGACTCCCAGTACG	Sense
JENS(6,270-6,289)	CTGTCAGTGTACTGAATGCC	Antisense
JENS(6,171-6,190)	GGAGAGTACCGTTCTAGAGG	Sense
JENS(7,041-7,060)	ACTGTGCTCCCCCCATACAG	Antisense
JENS(6,981-7,000)	CCGGATTGCCAAGCATGGCA	Sense
JENS(7,981-8,000)	CGCCCCACCTTTCGTGTACC	Antisense
JENS(7,921-7,940)	GCGGGCGCGGAAGCTGGAAC	Sense
JENS(8,931-8,950)	CAGGCCGTGCTCCATTGATT	Antisense
JENS(8,891-8,910)	CAACAGCAACGCGTCTCTCG	Sense
JENS(9,901-9,920)	TCCTGGGGAGATGCGCGCCC	Antisense
JENS(9,861-9,880)	CTACTCGTCCCGTGCAGAGG	Sense
JENS(10,944-10,963)	AGATCCTGTGTTCTTCCTCA	Antisense
